# Data on the activation and utilization of an electronic health record patient portal in an adult inpatient population at an academic medical center

**DOI:** 10.1016/j.dib.2021.106806

**Published:** 2021-01-30

**Authors:** Corey T. Allard, Matthew D. Krasowski

**Affiliations:** Department of Pathology, University of Iowa Hospitals and Clinics, 200 Hawkins Drive, Iowa City, IA 52242, USA

**Keywords:** Consumer health informatics, Diagnostic imaging, Electronic health records, Medical informatics, Minority groups, Patient portals, Proxy, User-computer interface

## Abstract

Electronic health record patient portals allow patients to access their own health data online and interact with the healthcare team. Many studies have focused on use of patient portals in the outpatient setting. Relatively less is known about use of patient portals for hospitalized patients. The data in this article include analysis of patient portal activation and utilization for adults hospitalized in 2018 at an academic medical center in a Midwestern state in the United States. Activation was assessed by percentage of patients who had activated their patient portal by the time of data review. Utilization of the patient portal was determined by whether patients or their legal proxies accessed one or more reports from diagnostic testing ordered during inpatient encounter(s) in 2018. The data include 826,843 diagnostic tests on 40,640 unique patients. Patient characteristics include sex, age, whether outpatient diagnostic tests were also performed in 2018, preferred language (English or non-English), health insurance status (private, public, or uninsured), self-declared race (White or non-White), and whether there was a legal proxy for the patient. Association of these covariates with patient portal activation and utilization were analyzed.

## Abstract (author highlights)

Value of the Data

• The data add to the existing limited published data on utilization of patient portals in the adult inpatient setting.
• Variations in portal usage by patient demographics provides opportunity for reducing health disparities.
• The data provides a baseline rate of portal usage prior to implementation of the federal mandate of the Cures Act in the United States.
• The data provide information for 826,843 diagnostic tests on 40,640 unique patients.

## Specifications Table

**Table:** 

Subject	Medicine and Dentistry
Specific subject area	Pathology and Medical Technology
Type of data	Tables Figures
How data were acquired	Retrospective data review from electronic health record data at an academic medical center.
Data format	Raw and Analyzed
Parameters for data collection	Retrospective data on all diagnostic testing performed on hospitalized adult patients at an academic medical center was obtained from the electronic health record (Epic, Inc.) covering the time period from January 1, 2018 through December 31, 2018. The data extraction also included whether the patients had an active patient portal (Epic MyChart) account by the time of data review (January 1, 2020) and whether specific diagnostic test reports were viewed in the patient portal. The project had approval from the University of Iowa Institutional Review Board.
Description of data collection	There were a total of 826,843 diagnostic test reports on 40,640 unique patients that were ordered during inpatient encounters. The data included whether the patient had an active eletronic health record patient portal account, whether the patient or proxy accessed the diagnostic test reports through the patient portal, and a number of demographic variables. The project was approved by the University of Iowa Institutional Review Board (protocol # 201907709) as retrospective analysis. The data collection also determined whether patients with inpatient encounters also had any diagnostic tests performed in the outpatient setting in 2018. Statistical analyses were performed using SPSS (PASW Statistics 18, Chicago, Illinois).
Data source location	Iowa City, Iowa, United States of America
Data accessibility	Repository: Mendeley data Data identification number: 10.17632/3wc6t92rrg.1 Direct URL to data: http://dx.doi.org/10.17632/3wc6t92rrg.1

## 1 Data Description

We compiled data on 826,843 diagnostic test (pathology/laboratory and radiology) reports from 40,460 unique patients that had testing performed during one or inpatient admission(s) at an academic medical center in 2018. There is growing literature on factors influencing patient engagement and use of electronic health record (EHR) patient portals, especially in the ambulatory setting [1], [2], [3], [4], [5], [6]. More recent research has focused on patient portal use in the acute care [7], emergency care [8], and inpatient settings [9], [10], [11], [12], [13], [14]. In the United States, a federal mandate known as the Cures Act will dictate that health systems release all medical notes and diagnostic test reports to patients when they are available [15]. Patient portals provide a major route to achieve these goals, and increased use will have significant impacts on patient engagement with their own healthcare [4,^11^,^16^,17]. The Cures Act was scheduled to take effect November 2, 2020 but has been delayed likely until spring of 2021. The data presented provide a baseline for inpatient portal use prior to any effects of the Cures Act.

Our institution (University of Iowa Hospitals and Clinics) adopted the Epic EHR for both inpatient and outpatient care in May 2009 and implemented MyChart in 2010 [8]. MyChart is a patient portal ‘tethered’ to the Epic EHR, meaning that MyChart allows patients (or legal proxies such as parents/guardians of young children) to access their own patient data from the Epic database once a password-protected account is set up [2,^4^,^5^,14]. Patients who are seen in either inpatient or outpatient settings within our institution are provided information to activate MyChart accounts. Once set up, the accounts can be viewed from either computers or mobile devices (including a smartphone app) that are connected to the internet. Patients can use MyChart to schedule appointments, view diagnostic test reports, renew prescriptions, fill out medical questionnaires or forms, access inpatient discharge summaries, and send messages to the healthcare team. The hardware hosting Epic and MyChart are maintained by Health Care Information Services of the University of Iowa. Hospital staff receive training in the functions of MyChart and often utilize MyChart in their own healthcare or that of their families. Specialized database reporting tools within Epic (described in more detail below) allow hospital staff to analyze patient utilization of MyChart, provided that ethical approval has been granted.

For the 40,460 unique patients that had diagnostic testing performed during hospitalization in 2018, account activation was assessed by percentage of patients who had activated their patient portal by the time of data review (January 2020). Utilization of the patient portal was determined by whether patients used the patient portal to access one or more reports from diagnostic tests ordered during hospitalization. During the retrospective time period, there were limited features of the patient portal for inpatient encounters. This included viewing of diagnostic test reports, discharge summaries, and post-visit summaries. Of these, only viewing of diagnostic test reports was captured as a metric in Epic that could be later analyzed. Hard copies of the post-visit summary were given to patients/families on discharge; this also contained information on activating a MyChart account for those who had not done so already.

Original data for this study are available at Mendeley Data (http://dx.doi.org/10.17632/3wc6t92rrg.1) which include data for 826,843 diagnostic test results on 40,640 unique patients. Specific data fields include: unique patient identification number (deidentified), number of inpatient admissions in 2018, whether test result was during the initial admission in 2018 or a subsequent admission, specific diagnostic test ordered, diagnostic test category, sex (as officially noted in the electronic health record), patient portal status (active or inactive), whether diagnostic test result was viewed in the patient portal, the most common self-declared race categories, simplified race category (White or non-White), age at time of testing, age category, primary/preferred language (as recorded in electronic health record; English on non-English), whether there was a legal proxy that had access to the patient's portal account, and insurance category (private/commercial or public/uninsured).

Table 1 shows statistics for viewing of diagnostic tests (pathology/laboratory and radiology), with utilization defined as viewing at least one test report by time of data review. The data is broken down by sex (male/female), whether outpatient diagnostics were also ordered in 2018, whether patient had only one or more than one inpatient admissions in 2018, preferred/primary language (English or non-English), health insurance status in 2018 (public insurance/uninsured or private insurance), self-declared race (White or non-White), and whether patient had legal proxy who could access the patient's EHR portal (establishing a proxy required committee approval and was typically due to patient mental incapacitation, intellectual disability, or other factors that impacted ability to make medical decisions). We employed univariate comparisons using Χ^2^ tests for categorical variables. We also performed multivariate logistic regression analysis to determine the association between patient medical and demographic characteristics and the endpoints of patient portal activation and utilization. On multiple logistic regression analysis, female sex, outpatient studies, performance of both laboratory and imaging studies during hospitalization, English as preferred language, commercial insurance, self-declared race as White, age, and presence of legal proxy for the patient showed significant association with MyChart account activation (Table 2) and viewing of diagnostic test reports (Table 3).

**Table 1:** Summary Data for Patient Population That Had Any Diagnostic Testing Performed in 2018

		Active Patient Portal Account					

	Number of Unique Patients	Total	At Least One Report Viewed	No Report Viewed	Inactive Patient Portal Account	Activation Significance^1^	Viewed vs. Not Viewed Significance^2^
Overall	40640	43.3%	16.9%	26.4%	56.7%	N/A	N/A
Female	21083	48.6%	20.2%	28.5%	51.4%	*p* < 0.0001	*p* < 0.0001
Male	19557	37.6%	13.3%	24.2%	62.4%		
No outpatient diagnostic tests in 2018	13452	32.5%	10.1%	22.4%	67.5%	*p* < 0.0001	*p* < 0.0001
Outpatient diagnostic tests in 2018	27188	48.7%	20.2%	28.4%	51.3%		
Only one inpatient admission in 2018	30083	40.6%	13.7%	26.9%	59.4%	*p* < 0.0001	*p* < 0.0001
Two or more admissions in 2018	10557	51.1%	26.0%	25.1%	48.9%		
Only lab or imaging tests done in 2018	29102	43.5%	14.5%	29.1%	56.5%	N.S. *p* = 0.13	*p* < 0.0001
Both lab and imaging tests in 2018	11538	42.7%	22.9%	19.8%	57.3%		
Primary language not English	1112	29.6%	9.4%	20.2%	70.4%	*p* < 0.0001	*p* < 0.0001
Primary language English	39528	43.7%	17.1%	26.6%	56.3%		
Public insurance/Uninsured	23394	36.8%	13.3%	23.5%	63.2%	*p* < 0.0001	*p* < 0.0001
Commercial insurance	17246	52.1%	21.7%	30.4%	47.9%		
Self-declared race not White	4948	33.0%	12.1%	20.8%	67.0%	*p* < 0.0001	*p* < 0.0001
Self-declared race White	35692	44.7%	17.5%	27.2%	55.3%		
No legal proxy for patient	39935	43.0%	16.6%	26.3%	57.0%	*p* < 0.0001	*p* < 0.0001
Legal proxy for patient	705	63.1%	29.9%	33.2%	36.9%		

1 Fisher exact t-test for number of patients with activated patient portal account compared to number of patients who have not activated patient portal accounts.

2 Fisher exact t-test for number of patients with viewed at least one report from diagnostic testing compared to number of patients who did not view any reports.

**Table 2:** Factors Affecting the Odds of MyChart Activation in Adult Inpatients

Variable	OR (95% CI)^1^	P^1^
Female sex	1.09 (1.08–1.10)	<.001
Outpatient studies in 2018	1.18 (1.17–1.19)	<.001
Multiple admissions in 2018	1.10 (1.09–1.11)	<.001
Both labs and imaging performed in 2018	1.03 (1.02–1.04)	<.001
Preferred language English	1.08 (1.05–1.11)	<.001
Commercial insurance	1.13 (1.12–1.14)	<.001
Self-declared race White	1.16 (1.14–1.18)	<.001
Age	0.995 (0.994–0.996)	<.001
Legal proxy for the patient	1.16 (1.12–1.21)	<.001

1 CI; confidence interval; OR, odds ratio. OR > 1.0 indicates increased odds of MyChart account activation. Analysis uses multivariate logistic regression analysis.

**Table 3:** Factors Affecting the Odds of MyChart Utilization (Viewing of Diagnostic Test Report) in Adult Inpatients

Variable	OR (95% CI)^1^	P^1^
Female sex	1.08 (1.05–1.10)	<.001
Outpatient studies in 2018	1.003 (1.003–1.003)	<.001
Multiple admissions in 2018	1.10 (1.07–1.13)	<.001
Both labs and imaging performed in 2018	1.09 (1.09–1.11)	<.001
Preferred language English	1.10 (1.09–1.11)	<.001
Commercial insurance	1.05 (1.2–1.07)	<.001
Self-declared race White	1.05 (1.05–1.06)	<.001
Age	1.08 (1.06–1.09)	<.001
Legal proxy for the patient	1.07 (1.07–1.08)	<.001

1 CI; confidence interval; OR, odds ratio. OR > 1.0 indicates increased odds of MyChart account activation. Analysis uses multivariate logistic regression analysis.

Table 4 uses the same dataset as Table 1 but with data expressed as percent viewing of total diagnostic tests. Table 5 uses the same format as Table 1 but with the dataset restricted to pathology/laboratory tests only. This table also includes statistics for whether patients who had pathology/laboratory tests also had radiology tests performed in 2018 during hospitalization. Table 6 uses the same dataset as Table 5 but with data expressed as percent viewing of total tests. Table 7 uses the same format as Table 1 but with the dataset restricted to imaging studies only. This table also includes statistics for whether patients who had radiology tests also had pathology/laboratory tests performed in 2018 during hospitalization. Table 8 shows the same dataset as Table 7 but with data expressed as percent viewing of total tests.

**Table 4:** Summary Data for Overall Accessing of Diagnostic Tests (Laboratory and Imaging Tests Combined)

	Number of Inpatient Diagnostic Tests	Active Patient Portal Account but Report Not Viewed	Inactive Patient Portal Account	Report Viewed	Viewed vs Not Viewed Significance^1^
Overall	826843	33.5%	60.2%	6.4%	N/A
Female	370292	34.1%	58.3%	7.6%	*p* < 0.0001
Male	456551	33.0%	61.7%	5.4%	
No outpatient diagnostic tests in 2018	178182	21.7%	74.6%	3.7%	*p* < 0.0001
Outpatient diagnostic tests in 2018	648661	36.7%	56.2%	7.1%	
First or initial admission in 2018	585177	31.1%	63.3%	5.7%	*p* < 0.0001
Second or later admissions in 2018	241666	39.3%	52.6%	8.1%	
Only labs or imaging tests in 2018	282334	32.7%	59.5%	7.8%	*p* < 0.0001
Both lab and imaging tests in 2018	544509	33.9%	60.5%	5.6%	
Primary language not English	22413	16.5%	80.1%	3.4%	*p* < 0.0001
Primary language English	804430	33.9%	59.6%	6.5%	
Public insurance/Uninsured	559629	28.6%	66.9%	4.4%	*p* < 0.0001
Commercial insurance	267214	43.6%	45.9%	10.5%	
Self-declared race not White	99437	23.5%	72.3%	4.2%	*p* < 0.0001
Self-declared race White	727406	34.8%	58.5%	6.7%	
No legal proxy for patient	814459	33.1%	60.7%	6.2%	*p* < 0.0001
Legal proxy for patient	12384	58.6%	25.1%	16.2%	

1 Chi square 2 × 2 with Yates’ correction for number of patients with viewed at least one report from diagnostic testing compared to number of patients who did not view any reports.

**Table 5:** Summary Data for Patient Population That Had Any Laboratory Tests Performed in 2018

		Active Patient Portal Account					
	Number of Unique Patients	Total	At Least One Report Viewed	No Report Viewed	Inactive Patient Portal Account	Activation Significance^1^	Viewed vs. Not Viewed Significance^2^
Overall	28420	45.6%	19.7%	25.9%	54.4%	N/A	N/A
Female	15221	51.3%	23.1%	28.2%	48.7%	*p* < 0.0001	*p* < 0.0001
Male	13199	38.9%	15.8%	23.1%	61.1%		
No outpatient diagnostic tests in 2018	8125	29.9%	11.2%	18.7%	70.2%	*p* < 0.0001	*p* < 0.0001
Outpatient diagnostic tests in 2018	20295	51.9%	23.1%	28.8%	48.2%		
Only one inpatient admission in 2018	21456	45.3%	17.8%	27.5%	54.7%	*p* < 0.0001	*p* < 0.0001
Two or more admissions in 2018	6981	49.4%	24.3%	25.1%	50.6%		
Only lab tests done in 2018	16877	47.5%	19.4%	28.1%	52.5%	*p* < 0.0001	N.S. *p* = 0.16
Both lab and imaging tests in 2018	11543	42.7%	20.1%	22.6%	57.3%		
Primary language not English	768	24.6%	8.7%	15.9%	75.4%	*p* < 0.0001	*p* < 0.0001
Primary language English	27652	46.1%	20.0%	26.1%	53.9%		
Public insurance/Uninsured	17403	36.1%	14.2%	21.9%	63.9%	*p* < 0.0001	*p* < 0.0001
Commercial insurance	11017	60.4%	28.2%	32.2%	39.6%		
Self-declared race not White	3500	35.4%	14.3%	21.1%	64.6%	*p* < 0.0001	*p* < 0.0001
Self-declared race White	24920	46.9%	20.4%	26.5%	53.1%		
No legal proxy for patient	27979	45.0%	19.3%	25.7%	55.0%	*p* < 0.0001	*p* < 0.0001
Legal proxy for patient	441	74.6%	39.2%	35.4%	25.4%		

1 Fisher exact t-test for number of patients with activated patient portal account compared to number of patients who have not activated patient portal accounts.

2 Fisher exact t-test for number of patients with viewed at least one report from diagnostic testing compared to number of patients who did not view any reports.

**Table 6:** Summary Data for Overall Accessing of Laboratory Tests

	Number of Inpatient Diagnostic Tests	Active Patient Portal Account but Report Not Viewed	Inactive Patient Portal Account	Report Viewed	Viewed vs Not Viewed Significance^1^
Overall	782410	29.9%	63.5%	6.5%	N/A
Female	348386	31.1%	61.0%	7.8%	*p* < 0.0001
Male	434024	29.0%	65.5%	5.5%	
No outpatient diagnostic tests in 2018	163082	18.9%	77.3%	3.7%	*p* < 0.0001
Outpatient diagnostic tests in 2018	619328	32.8%	59.9%	7.3%	
First or initial admission in 2018	553081	28.5%	65.7%	5.8%	*p* < 0.0001
Second or later admissions in 2018	229329	33.5%	58.2%	8.3%	
Only labs tests in 2018	266471	30.6%	61.5%	7.9%	*p* < 0.0001
Both lab and imaging tests in 2018	515939	29.6%	64.6%	5.8%	
Primary language not English	21175	14.7%	81.8%	3.5%	*p* < 0.0001
Primary language English	761235	30.4%	63.0%	6.6%	
Public insurance/Uninsured	532545	25.2%	70.3%	4.5%	*p* < 0.0001
Commercial insurance	249865	40.0%	49.2%	10.8%	
Self-declared race not White	93913	20.2%	75.5%	4.3%	*p* < 0.0001
Self-declared race White	688497	31.3%	61.9%	6.8%	
No legal proxy for patient	771100	29.7%	63.9%	6.4%	*p* < 0.0001
Legal proxy for patient	11310	46.7%	36.1%	17.3%	

1 Chi square 2 × 2 with Yates’ correction for number of patients with viewed at least one report from diagnostic testing compared to number of patients who did not view any reports.

**Table 7:** Summary Data for Patient Population That Had Any Imaging Studies Performed in 2018

		Active Patient Portal Account					
	Number of Unique Patients	Total	At Least One Report Viewed	No Reports Viewed	Inactive Patient Portal Account	Activation Significance^1^	Viewed vs. Not Viewed Significance^2^
Overall	23758	43.4%	10.5%	32.9%	56.6%	N/A	N/A
Female	12583	49.6%	14.0%	35.6%	50.4%	*p* < 0.0001	*p* < 0.0001
Male	11175	36.3%	6.6%	29.7%	63.7%		
No outpatient diagnostic tests in 2018	8535	41.0%	9.5%	31.5%	59.0%	*p* < 0.0001	*p* < 0.0001
Outpatient diagnostic tests in 2018	15223	44.7%	11.1%	33.6%	55.3%		
Only one inpatient admission in 2018	18122	43.4%	8.0%	35.4%	56.6%	*p* < 0.0001	*p* < 0.0001
Two or more admissions in 2018	5636	66.4%	12.4%	54.0%	33.6%		
Only lab or imaging tests done in 2018	8535	47.5%	19.4%	28.1%	52.5%	*p* < 0.0001	*p* < 0.0001
Both lab and imaging tests in 2018	15223	42.7%	20.1%	22.6%	57.3%		
Primary language not English	645	42.0%	10.5%	31.5%	58.0%	N.S. *p* = 0.51	N.S. *p* = 1.0
Primary language English	23113	43.4%	10.5%	32.9%	56.6%		
Public insurance/Uninsured	13651	44.5%	10.6%	33.9%	55.5%	*p* < 0.0001	N.S. *p* = 0.48
Commercial insurance	10106	41.9%	10.4%	31.5%	58.1%		
Self-declared race not White	2944	31.4%	7.0%	24.4%	68.6%	*p* < 0.0001	*p* < 0.0001
Self-declared race White	20814	45.0%	11.0%	34.0%	55.0%		
No legal proxy for patient	23274	43.3%	10.5%	32.8%	56.7%	*p* < 0.0001	*p* < 0.0001
Legal proxy for patient	484	44.8%	11.8%	33.0%	55.2%		

1 Fisher exact t-test for number of patients with activated patient portal account compared to number of patients who have not activated patient portal accounts.

2 Fisher exact t-test for number of patients with viewed at least one report from diagnostic testing compared to number of patients who did not view any reports.

**Table 8:** Summary Data for Overall Accessing of Imaging Study Reports

	Number of Inpatient Imaging Studies	Active Patient Portal Account but Report Not Viewed	Inactive Patient Portal Account	Report Viewed	Viewed vs Not ViewedSignificance^1^
Overall	44433	52.4%	41.2%	6.4%	N/A
Female	29929	60.1%	32.9%	6.9%	*p* < 0.0001
Male	14504	36.3%	58.3%	5.4%	
No outpatient diagnostic tests in 2018	15099	49.5%	44.2%	6.2%	N.S. *p* = 0.29
Outpatient diagnostic tests in 2018	29334	53.8%	39.7%	6.5%	
First or initial admission in 2018	32096	45.2%	48.4%	6.4%	N.S. *p* = 0.77
Second or later admissions in 2018	12337	71.0%	22.5%	6.5%	
Only imaging tests in 2018	15863	37.8%	56.1	6.1%	N.S. *p* = 0.05
Both lab and imaging tests in 2018	28750	60.5%	32.9%	6.6%	
Primary language not English	1246	54.0%	39.8%	6.2%	N.S. *p* = 0.78
Primary language English	22600	52.3%	41.3%	6.4%	
Public insurance/Uninsured	27192	54.4%	39.5%	6.1%	*p* = 0.0001
Commercial insurance	17421	49.3%	43.8%	6.9%	
Self-declared race not White	5749	42.1%	53.8%	4.1%	*p* < 0.0001
Self-declared race White	38684	53.9%	39.3%	6.8%	
No legal proxy for patient	43359	52.2%	41.3%	6.4%	N.S. *p* = 0.67
Legal proxy for patient	1074	58.2%	35.8%	6.1%	

1 Chi square 2 × 2 with Yates’ correction for number of patients with viewed at least one report from diagnostic testing compared to number of patients who did not view any reports.

**Fig. 1:**
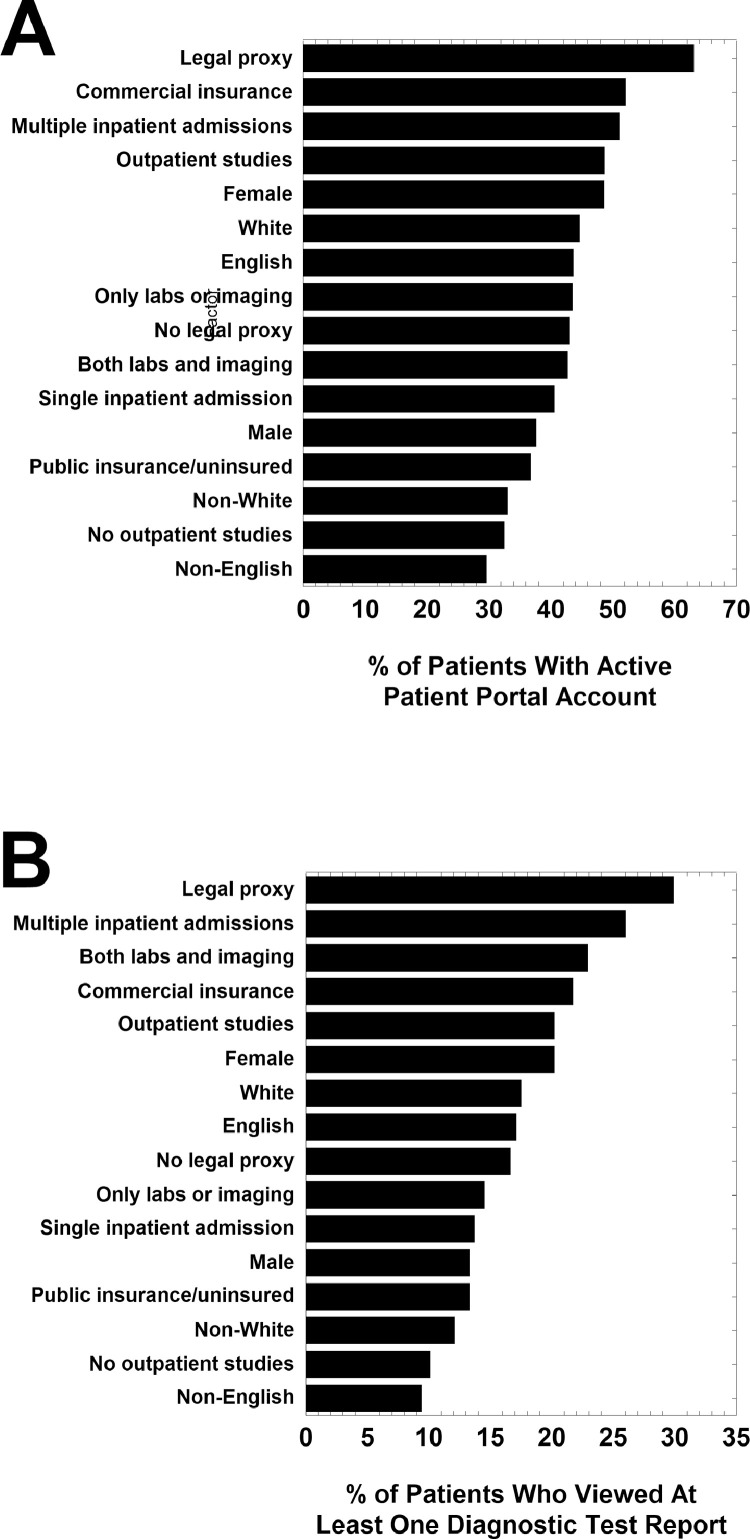
Patient portal (A) activation and (B) view rates of diagnostic test reports by percentage of patients by subcategories. View rates indicate whether patient or proxy viewed at least one report from diagnostic testing ordered during hospitalization in 2018.

**Fig. 2:**
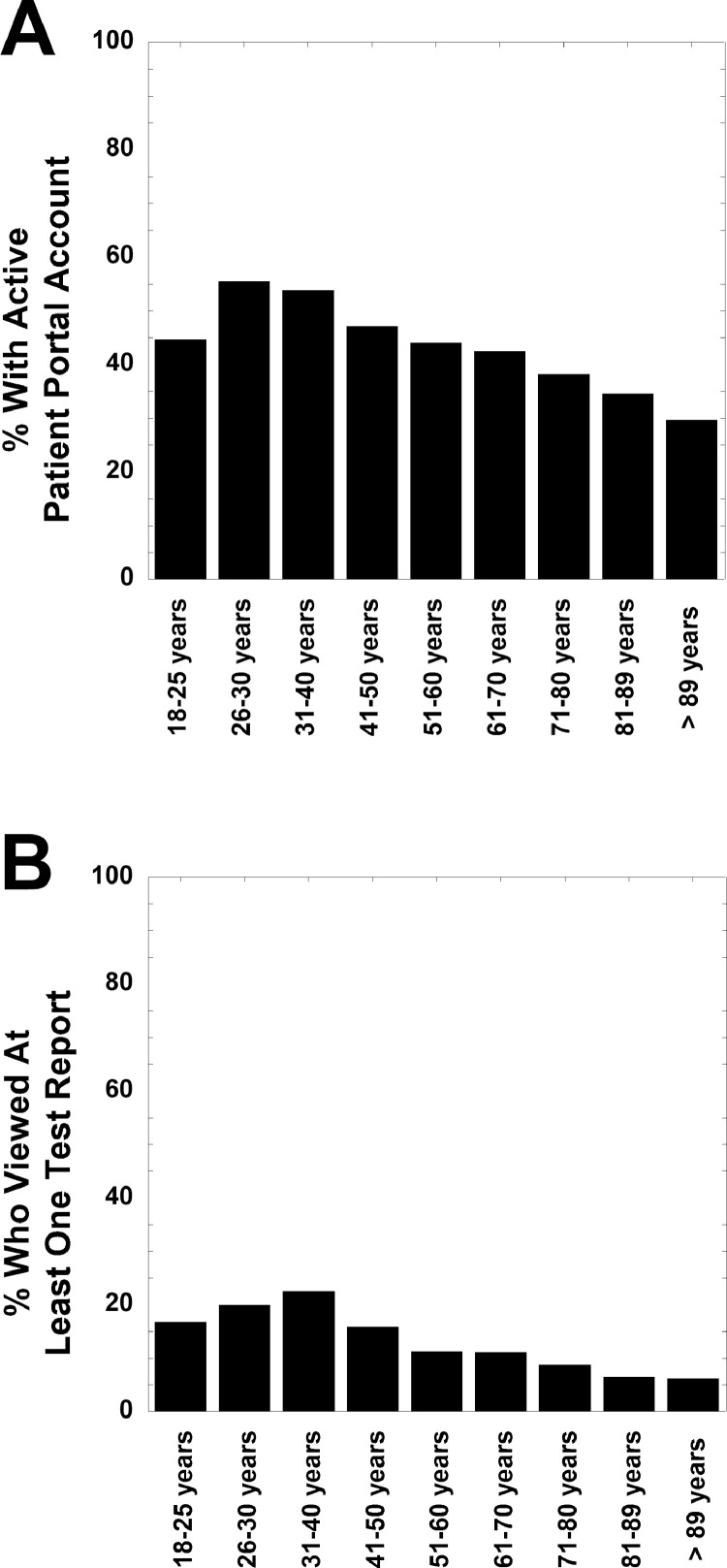
Percent of patients within various age brackets who have (A) active patient portal account and (B) viewed at one report from diagnostic testing performed during hospitalization(s) in 2018.

**Fig. 3:**
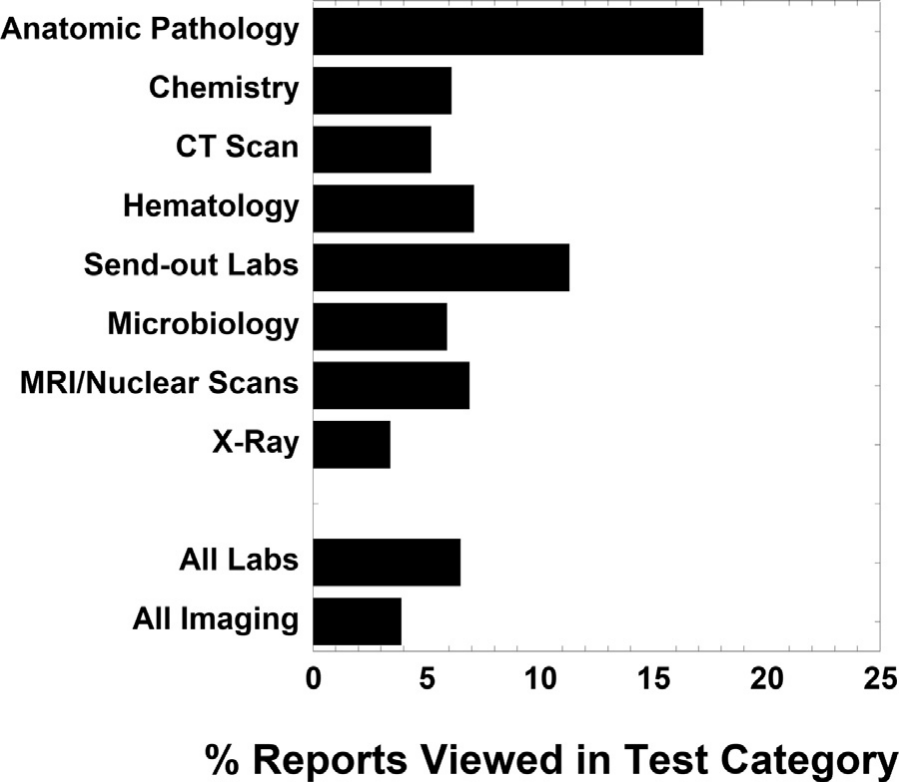
Percent of all diagnostic test reports viewed in various test categories.

Fig. 1 displays the percent of patients within various subcategories who have active patient portal account (panel A) and who viewed at least one diagnostic test reports (pathology/laboratory and radiology) performed during hospitalization in 2018 (panel B). This figure uses the entire dataset of pathology/laboratory and radiology tests summarized in Table 1. Fig. 2 displays the percent of patients within various age brackets who have active patient portal account (panel A) and who viewed at least one diagnostic test report (pathology/laboratory and radiology) performed during hospitalization in 2018 (panel B). Fig. 3 shows the percent of all diagnostic test reports viewed in various test categories. The test categories are Anatomic Pathology (which includes surgical pathology, cytopathology, and dermatopathology), Chemistry (includes clinical chemistry, therapeutic drug monitoring, and toxicology), computed tomography (CT) scan, Hematology (includes bone marrow and hemostasis/thrombosis), Send-out Tests (sent to reference laboratory), Microbiology, magnetic resonance imaging (MRI)/nuclear scans, and X-rays. Data is also aggregated as All Labs (pathology/laboratory) and All Imaging.

## 2 Experimental Design Materials and Methods

Epic Reporting Workbench (RWB), a software tool for retrieving specific data from the EHR, was used to retrieve all diagnostic test orders (pathology/laboratory and radiology) ordered during hospitalization between January 1, 2018 and December 31, 2018 on patients 18 years and older [18]. With each diagnostic test order, the RWB search also retrieved age, sex (male or female), self-identified race, self-identified preferred language, insurance status, prior hospitalization, presence of outpatient lab reports, and whether patient had a proxy that could legally access their patient portal. Race was categorized as White or race other than White (included African-American, American Indian / Alaska Native, Asian, Hispanic/Latino, Mixed Race, Native Hawaiian / Pacific Islander, Other, Unknown and Declined), language as English or Non-English (included a total of 75 other languages with the six most common other than English being Spanish, Arabic, French, Chinese, Swahili and Vietnamese) and insurance as commercial payor or public insurance (Medicare or Medicaid)/uninsured.

Data was also analyzed by testing category. Pathology/laboratory tests were categorized as Anatomic Pathology (including surgical biopsies and resections, cytopathology, and dermatopathology), Chemistry (includes toxicology, therapeutic drug monitoring, and clinical chemistry), Hematology, Microbiology, and Send-out (referred to outside reference laboratories). Imaging tests were categorized as computerized tomography (CT) scan, magnetic resonance imaging (MRI), nuclear scans, and X-rays.

## Ethics Statement

The analyses had approval by the University of Iowa Institutional Review Board (protocol # 201907709) as a retrospective project.

## CRediT Author Statement

**Corey Allard:** Formal analysis, Writing – Review & Editing, Visualization; **Matthew Krasowski:** Formal analysis, Conceptualization, Writing – Original Draft, Writing – Review & Editing, Methodology, Supervision.

## Declaration of Competing Interest

The authors declare they have no known competing financial interests or personal relationships which have, or be perceived to have, influenced the work in this article.
